# Expression Profiles of Genes Encoding Cornified Envelope Proteins in Atopic Dermatitis and Cutaneous T-Cell Lymphomas

**DOI:** 10.3390/nu12030862

**Published:** 2020-03-24

**Authors:** Magdalena Trzeciak, Berenika Olszewska, Monika Sakowicz-Burkiewicz, Małgorzata Sokołowska-Wojdyło, Jerzy Jankau, Roman Janusz Nowicki, Tadeusz Pawełczyk

**Affiliations:** 1 Department of Dermatology, Venereology and Allergology, Medical University of Gdansk, 80-214 Gdansk, Poland; mtrzeciak@gumed.edu.pl (M.T.);; 2 Department of Molecular Medicine, Medical University of Gdansk, 80-214 Gdansk, Poland; 3 Department of Plastic Surgery, Medical University of Gdańsk, 80-214 Gdańsk, Poland

**Keywords:** mycosis fungoides, atopic dermatitis, cutaneous lymphomas, cornified envelope proteins, FLG

## Abstract

The skin barrier defect in cutaneous T-cell lymphomas (CTCL) was recently confirmed to be similar to the one observed in atopic dermatitis (AD). We have examined the expression level of cornified envelope (CE) proteins in CTCL, AD and healthy skin, to search for the differences and their relation to the courses of both diseases. The levels of *FLG, FLG2, RPTN, HRNR, SPRR1A, SPRR1B, SPRR3* and *LELP-1* mRNA were determined by qRT-PCR, while protein levels were examined using the ELISA method in skin samples. We have found that mRNA levels *of FLG, FLG2, LOR, CRNN* and *SPRR3v1* were decreased (*p* ≤ 0.04), whereas mRNA levels of *RPTN, HRNR* and *SPRR1Av1* were increased in lesional and nonlesional AD skin compared to the healthy control group (*p* ≤ 0.04). The levels of *FLG, FLG2, CRNN, SPRR3v1* mRNA increased (*p* ≤ 0.02) and *RPTN, HRNR* and *SPRR1Av1* mRNA decreased (*p* ≤ 0.005) in CTCL skin compared to the lesional AD skin. There was a strong correlation between the stage of CTCL and increased *SPRR1Av1* gene expression at both mRNA (R = 0.89; *p* ≤ 0.05) and protein levels (R = 0.94; *p* ≤ 0.05). FLG, FLG2, RPTN, HRNR and SPRR1A seem to play a key role in skin barrier dysfunction in CTCL and could be considered a biomarker for differential diagnosis of AD and CTCL. *SPRR1Av1* transcript levels seem to be a possible marker of CTCL stage, however, further studies on a larger study group are needed to confirm our findings.

## 1. Introduction

Cutaneous T-cell lymphomas (CTCLs) represent a rare heterogeneous group of extranodal non-Hodgkin lymphomas. CTCLs are characterized by an infiltration of the skin with neoplastic CD4 + CD45RO + skin-homing T-cells [1,2]. The pathogenesis of CTCL remains unexplained. There are a few hypotheses, including chronic antigen stimulation, viral or bacterial, leading to a loss of immune-surveillance and, therefore, the proliferation of neoplastic T-cells. Moreover, chromosomal instability and abnormal expression of genes involved in the cell cycle and a complex series of interactions between different cells in skin microenvironment have been suggested [3,4,5,6]. The inflammatory microenvironment of the skin seems to play a crucial role in CTCL pathogenesis, as the predominance of Th2 over Th1 cells in inflammatory microenvironment seems to be responsible for the suppression of antitumor response, proliferation of malignant cells and escape from immunosurveillance [7,8,9]. The clinical spectrum of CTCL varies widely; the predominant subtype is mycosis fungoides (MF), accounting for 60% [1]. The clinical presentation of MF follows some clinical stages, ranging from patch-like lesions in the early stage, through plaque stage, erythroderma and tumors; usually, all stages are accompanied by severe pruritus [1]. MF is also a significant diagnostic challenge in histopathological examination as it may mimic many benign dermatoses such as atopic dermatitis (AD) [1,2]. CTCL shares many clinical and histological characteristics with AD. Not only the clinical presentation but also immunological features are overlapping. Both MF and AD show infiltration of the skin by skin-homing T-cells [10,11]. The skin of MF patients similar to AD shows an impaired skin barrier with decreased expression of filaggrin, loricrin and antimicrobial peptides in the skin, which results in increased susceptibility towards *Staphylococcus aureus* colonization and infections [11,12]. The malignant T-cells were reported to be responsible for changes in the epidermis in CTCL and impaired epidermal barrier function [13]. Therefore, skin infections have been a significant clinical problem both in AD and in CTCL as a result of compromised skin barrier and a progressive immunodeficiency in CTCL. In fact, patients with progressive CTCL die more frequently from infection rather than from the disease itself [14]. Changes in the expression of cornified envelope proteins in the skin were shown to impact the development and course of AD [15,16,17]. In the face of so many similarities between CTCL and AD, evaluation of expression of genes encoding the cornified envelope (CE) proteins such as the late cornified envelope-like proline-rich protein-1 (LELP-1), small proline-rich proteins (SPRR1A, SPRR1B, SPRR3), repetin (RPTN), cornulin (CRNN), hornerin (HRNR), loricrin (LOR) and filaggrin (FLG, FLG2) might provide accurate information and findings to differentiate those diseases and determine their relation to the course and development of both diseases. 

## 2. Materials and Methods 

### 2.1. Patients and Samples

The study group included: 11 patients with AD (seven males, four females, median age 30, age range 14–59) and nine CTCL patients (eight males 8, females 1, median age 65, age range 26–75) treated at the Department of Dermatology, Venerology and Allergology of the Medical University of Gdańsk. CTCL patients were diagnosed according to the International Society of Cutaneous Lymphoma (ISCL) and the European Organization of Research and Treatment of Cancer (EORTC) criteria [18], while the diagnosis of AD was based on Hanifin and Rajka criteria [19]. The control group comprised 11 healthy volunteers (one male, 10 females, median age 34, age range 20–54), without a medical history of immunological diseases, allergies or malignancies. Punch biopsies for gene expression and protein level analysis were obtained from CTCL patients (nine from lesional skin), AD patients (11 from lesional skin and 11 from nonlesional skin) and healthy controls (11 from healthy skin). 

The study was approved by the Ethics Committee of the Medical University of Gdańsk (NKBBN/317/2018) and was conducted according to the principles of the Declaration of Helsinki. All participants signed an informed consent prior to any study procedure. 

### 2.2. Isolation of Total RNA from Skin Fragments

Isolation of total RNA was carried out in accordance with the Chomczyński procedure, with our own modifications [20]. The skin fragments (4-mm punch biopsies) were homogenized in a sterile tube with 1 mL of TRI reagent (Sigma-Aldrich, Poznań, Poland). Next, chloroform (250 μL) was added, and the samples were vigorously shaken, incubated at 4 °C for 15 min and centrifuged (10,000× *g* for 15 min at 4 °C). The upper aqueous phase was removed into a new Eppendorf tube where an equal volume of isopropanol was added, and RNA was precipitated by overnight incubation at −20 °C, followed by centrifugation (10,000× *g* for 15 min at 4 °C). RNA pellets were washed first with 96% and then with 75% (*v*/*v*) ethanol, air-dried, resolved in diethyl-pyrocarbonate-treated thermo-sterilized water (30 μL) and stored at −20 °C until further analysis.

### 2.3. Relative Quantitative Real-Time RT-PCR Analysis

*FLG, FLG2, RPTN, HRNR, LELP-1, SPRR 1A, SPRR1B, SPRR3*, *LOR* and *HRNR* mRNA levels were analyzed by real-time PCR (RT-PCR) with TaqMan primer–probe sets using the Path-ID Multiplex One-Step RT-PCR kit (Path-ID Multiplex One-Step RT-PCR Kit, Applied Biosystems, Foster City, CA, USA). The reference transcript (*ACTB* or *TBP* or *G6PD*) was used as an internal standard and was amplified together with each target gene transcript in the same way using primers and probes (Table 1). Data analysis was performed using LightCycler 480 II software (Roche Diagnostics International Ltd., Rotkreuz, Switzerland).

### 2.4. Protein Level Determination

Protein levels of FLG, FLG2, LELP-1, SPRR1A and SPRR1B were determined using an enzyme-linked immunoabsorbent assay (ELISA) standard kit (Antibody-Protein ELISA kit, MyBioSource Inc., San Diego, CA, USA). We analyzed 4 CTCL, 6 AD and 11 control group samples with ELISA. Skin specimens were weighed, rinsed with cold phosphate-buffered saline (PBS) and homogenized in PBS containing 0.5% Igepol CA-630 (1:2) on ice. The homogenates were subjected to three freeze–thaw cycles to break the cell membranes, and the homogenates were centrifuged for 5 min at 5000× *g*. The supernatants obtained were used for protein determination by ELISA kit according to the manufacturer’s protocol. The quantity of investigated protein in the sample was interpolated from the standard curve and corrected for dilution. 

### 2.5. Statistical Analysis 

Statistical analysis was carried out using the Mann–Whitney U test. A comparison between lesional and nonlesional skin from the same patient was performed with the Wilcoxon matched-pairs test (marked in figures by dashed line). Associations between the two variables were assessed based on Spearman’s rank correlation. The results were considered statistically significant when *p* < 0.05. Statistical analyses were performed using Statistica 13.3.

## 3. Results

### 3.1. mRNA Level of Cornified Envelop Proteins

Expression levels of almost all genes encoding the CE proteins differed significantly between the AD and control groups, except for *SPRR1B* and *LELP1.* We have found significantly lower levels of *FLG, FLG2, LOR* and *CRNN* mRNA in lesional and nonlesional AD skin compared to the healthy control group (*p* ≤ 0.02). mRNA level of *SPRR3v1* was also decreased in AD samples, however, only in lesional skin (*p* ≤ 0.00008). Moreover, mRNA levels of those genes were significantly decreased in lesional skin in comparison to the nonlesional skin of AD patients (*p* ≤ 0.04), except for CRNN. On the other hand, mRNA levels of *LELP1, RPTN, HRNR and SPRR1Av1* were significantly higher in AD skin than healthy controls (*p* ≤ 0.05). The lesional skin of AD patients demonstrated significantly higher expression of *HRNR* and *SPRR1Av1* mRNA in comparison to nonlesional AD skin samples (*p* ≤ 0.04). 

**Figure 1:**
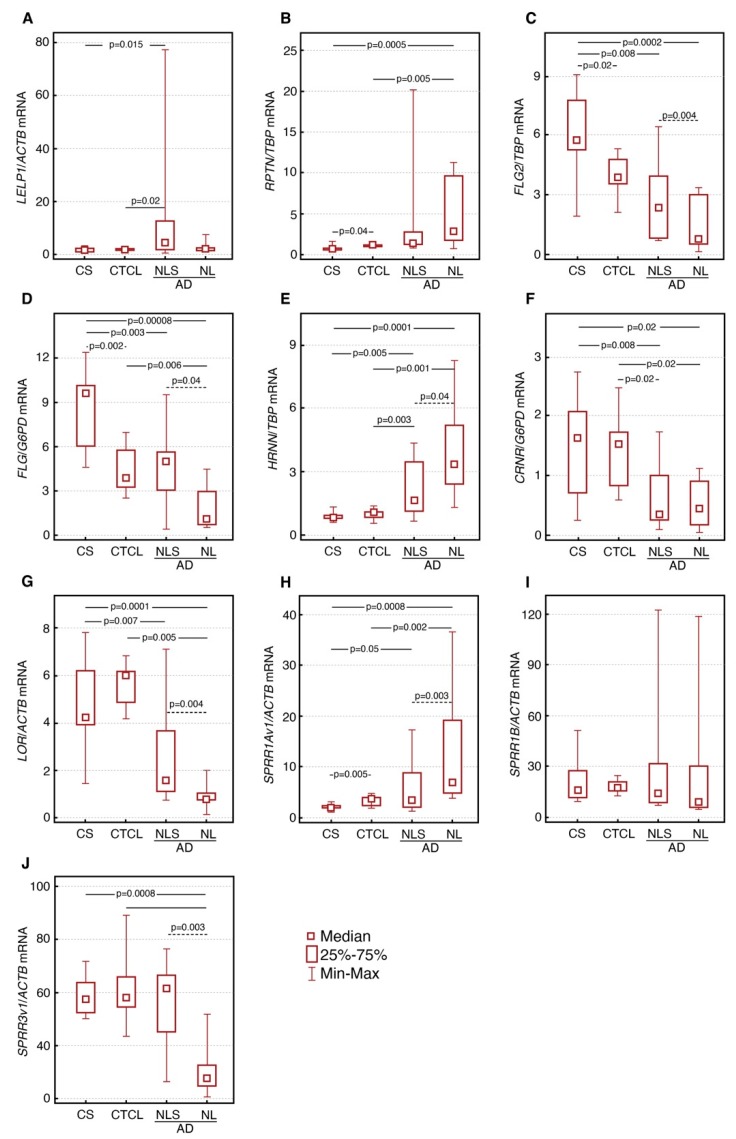
Levels of *LELP1* (**A**)*, RPTN* (**B**), *FLG2* (**C**), *FLG* (**D**)*, HRNR* (**E**), *CRNN* (**F**)*, LOR* (**G**)*, SPRR1Av1* (**H**)*, SPRR1B* (**I**) and *SPRR3v1* (**J**) transcripts in skin of healthy subjects (CS), cutaneous T-cell lymphomas (CTCL), lesional atopic dermatitis (LS AD) and nonlesional AD (NLS AD).

Differences in expression levels of genes encoding the CE proteins between CTCL skin and control group were observed only in a few genes. The mRNA levels of *FLG* and *FLG2* were significantly lower in CTCL skin compared to control skin (*p* ≤ 0.02), whereas *RPTN* and *SPRR1Av1* mRNA expression were significantly higher (*p* ≤ 0.04). All CE genes mRNA levels in skin of CTCL, AD and healthy subjects are demonstrated in detail in Figure 1.

Except for *LELP1*, *SPRR1B* and *SPRR3*, all other CE genes showed significant differences in mRNA expression between CTCL and both lesional and nonlesional AD skin samples. We observed that the levels of *LOR* and *CRNN* mRNA were significantly increased and *HRNR* mRNA was decreased in CTCL skin compared to both lesional and nonlesional AD skin. The levels of *FLG, FLG2* and *SPRR3v1* mRNA were significantly higher in CTCL than lesional AD skin. Moreover, *RPTN*, *HRNR* and *SPRR1Av1* mRNA expression were significantly lower in CTCL skin compared to the lesional AD skin.

### 3.2. Cornified Envelope Proteins Levels

**Figure 2:**
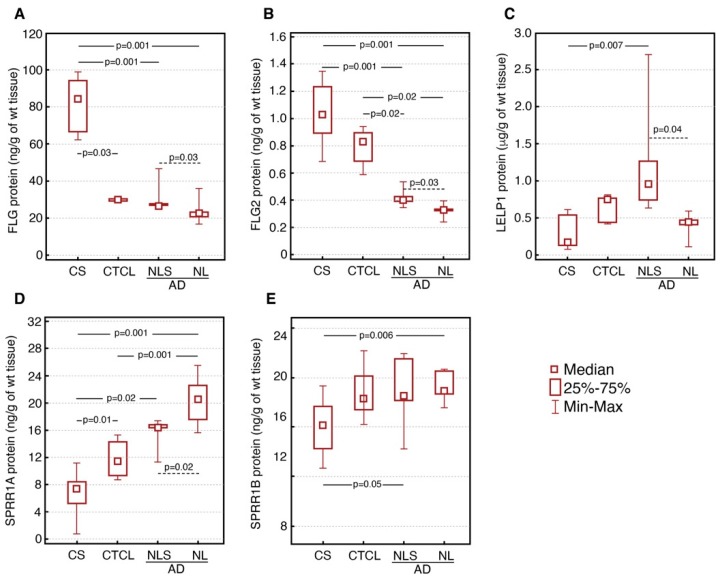
Levels of FLG (**A**), FLG2 (**B**), LELP1 (**C**), SPRR1A (**D**) and SPRR1B (**E**) proteins in skin of healthy subjects (CS) and patients with CTCL, lesional AD (LS AD) and nonlesional AD (NLS AD).

**Figure 3:**
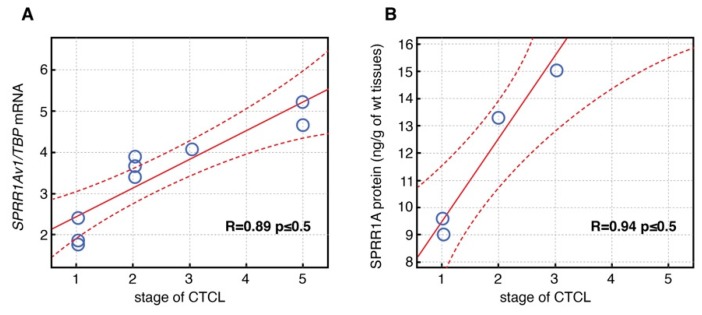
Correlation of *SPRR1Av1* gene expression at both mRNA (**A**) and protein levels (**B**) with stage of disease.

The levels of FLG and FLG2 proteins were significantly lower in CTCL and both lesional and nonlesional AD skin compared to healthy controls skin. Both FLG and FLG2 levels were significantly lower in lesional than nonlesional AD skin samples. The level of FLG2 protein was increased in CTCL compared to AD skin samples (*p* ≤ 0.02). SPRR1A protein level both in CTCL (*p* ≤ 0.01) and AD skin (*p* ≤ 0.001) was significantly increased in comparison with healthy skin. Moreover, the level of SPRR1A in CTCL was lower than in lesional (*p* ≤ 0.02) and nonlesional AD skin (*p* ≤ 0.025). On the other hand, the level of SPRR1B was distinctly higher in AD lesional and nonlesional skin samples compared with the skin of healthy subjects. Levels of FLG, FLG2, LELP1, SPRR1A and SPRR1B proteins in skin of CTCL, AD and healthy subjects are demonstrated in Figure 2. We have also found a significant correlation between increased *SPRR1Av1* gene expression at both mRNA (R = 0.89; *p* ≤ 0.05) and protein levels (R = 0.94; *p* ≤ 0.05) and stage of disease (Figure 3).

## 4. Limitations of This Study

We found some discrepancies between our finding and those of and other studies [11] that might be related to our relatively small study group with only a few cases of advanced-stage CTCL, which is a limitation of our study. Moreover, the major limitation is the disparity in the number of samples from female and male volunteers between the control, CTCL and AD groups. Therefore, it would be beneficial to perform further studies that would include a larger study group and a suitable number of female and male patients.

## 5. Discussion

Factors such as cornified envelope proteins that contribute to skin barrier dysfunction have been extensively studied, and their role in AD skin barrier defects has been proven [15,16,17,21,22,23,24]. However, reports concerning their role in CTCL are rather scarce. There is only one study, by Suga et al., that has analyzed skin barrier dysfunction in CTCL [11]. Decreased levels of *FLG* and *LOR* mRNA have already been demonstrated in the skin of CTCL and psoriasis patients, which confirms that skin barrier dysfunction is not specific to AD pathogenesis [11]. Due to some similarities between CTCL and AD, CE proteins appear to be a promising target for markers helpful in differential diagnosis of those diseases. 

Our study has revealed decreased mRNA expression of *FLG, FLG2, LOR, CRNN* and *SPRR3v1* concomitant with increased *LELP1, RPTN, HRNR* and *SPRR1Av1* mRNA levels in AD compared to healthy controls, which is in line with previous findings [15,16,17,21,22,23,24]. Moreover, the majority of examined CE proteins were decreased in comparison to healthy skin, which confirms that pathological changes also occur in nonlesional AD skin. We have noticed similar upward and downward tendencies in the expression of genes encoding some of the CE proteins in AD and CTCL skin in comparison to healthy controls. However, alterations in *HRNR, SPRR3v1, LOR* and *CRNN* mRNA levels in skin are more specific for AD than CTCL. Despite similarities in CE protein expression, the same significant differences between CTCL and AD have also been demonstrated. 

We observed that trends of mRNA expression of *LELP1, HRNR, CRNN, LOR, SPRR1B* and *SPRR3* in CTCL were consistent with expression in the control group rather than the AD group, indicating that those CE proteins do not participate in the CTCL skin barrier defect. It seems that FLG, FLG2, RPTN and SPRR1A are the true key players in CTCL skin barrier deficiency, as levels of their transcripts differed significantly in comparison to healthy controls. Moreover, mentioned levels of mRNA expression in skin and levels of FLG2 and SPRR1A proteins differed significantly between both AD and healthy subjects. FLG, FLG2, RPTN and SPRR1A together might be considered potential candidate biomarkers for differential diagnosis in AD and CTCL using either PCR or ELISA method. 

Our findings concerning CTCL are partially in line with previous study results [11]. Filaggrin mRNA expression levels in skin of CTCL patients were significantly lower than in the control group, which is in accordance with the findings of Suga et al. [11]. However, *LOR* mRNA expression did not differ between CTCL and healthy skin, which is contrary to the previous report [11]. 

Recently, Kim et al. demonstrated that Th2 cytokines such as IL-4 and IL-13 inhibit expression of filaggrin and loricrin by primary human keratinocytes [25]. It seems that the immunity against infections is downregulated not only by a predominance of Th2 cytokine pattern in CTCL skin microenvironment but also by the skin barrier dysfunction. It would be worth examining whether expression of other CE proteins is also downregulated by Th2 cytokines. Furthermore, the hypothesis of CTCL pathogenesis, concerning chronic antigen stimulation with bacterial superantigens such as *S. aureus* leading to the development and clonal proliferation of malignant T-cells seems to be up to date [4,26,27,28,29]. In the resent study, Lindahl and coworkers reported inhibition of malignant T-cells and clinical improvement in patients with advanced-stage CTCL after antibiotic treatment [29]. It appears that CTCL progression is powered by bacteria. Therefore, the skin barrier seems to be essential in disease activity.

SPRR proteins were demonstrated to have protective functions, and their overexpression was associated with exposure to factors such as ultraviolet radiation [30]. Moreover, SPRR1 was demonstrated to be overexpressed in severe rather than moderate AD, suggesting compensation of other CE protein deficiencies [15,21]. We have noted a positive correlation between *SPRR1Av1* mRNA expression and the stage of disease, which is in line with previous findings indicating its remedial functions [15]. We have also observed significantly higher expression of *RPTN* mRNA in CTCL than in the control group. It could be speculated that overexpression of RPTN as of SPRR1Av1 compensates filaggrin deficiency; however, this needs further study. 

According to the presented results, it seems that certain cornified envelope proteins play a key role in skin barrier dysfunction both in CTCL and AD. Moreover, differences in protein expression indicate that cornified envelope proteins might be considered biomarkers for differential diagnosis of AD and CTCL, especially in early-stage MF, when the differentiation even in histopathological examination is challenging. Skin barrier dysfunction is a well-known reason for AD development and exacerbation of the disease. Our results provide evidence that the skin barrier is also essential in the course of CTCL, probably contributing to the progression of the disease. The basic treatment for atopic dermatitis is the restoration of the skin barrier with emollients, which not only decreases transepidermal water loss and repairs the epidermal barrier but also prevents AD exacerbation, skin infections and AD development in infant AD risk groups [31,32]. Moreover, restoration of skin barrier functions indirectly decreases inflammation [33]. Based on our results, it seems that the approach to the CTCL treatment should be revised and complemented with the restoration of epidermal barrier functions by the usage of emollients.

In conclusion, we have demonstrated that skin barrier deficiency is not unique to AD, but it is also present in CTCL. Skin barrier dysfunction underlines AD development and course of disease, whereas in CTCL it rather contributes to the exacerbation of immunological processes due to skin infections. Only FLG, FLG2, RPTN, HRNR and SPRR1A proteins appear to be significant in skin barrier dysfunction. Moreover, they might be considered potential markers in difficult differential diagnosis of AD and CTCL. 

## Figures and Tables

**Table 1:** Gene transcript, primers and TaqMan probes used for real-time polymerase chain reaction (RT-PCR).

Gene Transcript	Primers	TaqMan Probe	Transcript of Reference Gene
*FLG* NM_002016.1	(F) ggactctgagaggcgatctg (R) tgctcccgagaagatccat	Universal ProbeLibrary Probe #38 (Roche)	Universal ProbeLibrary Reference Gene Assay Roche, Human *TBP* Gene Assay
*FLG2* NM_001014342.2	(F) tgactatggcctgcaacaa (R) ctttgaccctgaagctttgc	Universal ProbeLibrary Probe #73 (Roche)	Universal ProbeLibrary Reference Gene Assay Roche, Human *G6PD* Gene Assay
*LELP1* NM_001010857.1	(F) cccaagtgtgaacaaaagtg (R) ttcgaaacagcgttgcag	Universal ProbeLibrary Probe #26	Universal ProbeLibrary Reference Gene Assay Roche, Human *ACTB* Gene Assay
*SPRR1Avar.1* NM_001199828.1	(F) tcgggtgcatttgaggat (R) aaggaagactagggatggttca	Universal ProbeLibrary Probe #60 (Roche)	Universal ProbeLibrary Reference Gene Assay Roche, Human *ACTB* Gene Assay
*SPRR1B* NM_003125.2	(F) gagagacttaagatgaaagcaatga (R) tgaaagtgaatttaatgggggta	Universal ProbeLibrary Probe #24 (Roche)	Universal ProbeLibrary Reference Gene Assay Roche, Human *ACTB* Gene Assay
*SPRR3var.1* NM_005416.2	(F) tcaggagcttagaggattcttca (R) ttctgctggtaagaactcatgc	Universal ProbeLibrary Probe #89 (Roche)	Universal ProbeLibrary Reference Gene Assay Roche, Human *ACTB* Gene Assay
*RPTN* NM_001122965.1	(F) gctcttggctgagtttggag (R) aggttcaagatggtttccaca	Universal ProbeLibrary Probe #65 (Roche)	Universal ProbeLibrary Reference Gene Assay Roche, Human *TBP* Gene
*HRNR* NM_001009931.2	(F) caggggcaagatgggtattc (R) ccagaacttccccctcat	Universal ProbeLibrary Probe #69 (Roche)	Universal ProbeLibrary Reference Gene Assay Roche, Human *TBP* Gene Assay
*CRNN* NM_016190.2	(F) tggtcaaagatggatgcaag (R) cctgtcctcccggtactgt	Universal ProbeLibrary Probe #51 (Roche)	Universal ProbeLibrary Reference Gene Assay Roche, Human *G6PD* Gene
*LOR* NM_000427.2	(F) ctcacccttcctggtgctt (R) gaggtcttcacgcagtcca	Universal ProbeLibrary Probe #12 (Roche)	Universal ProbeLibrary Reference Gene Assay Roche, Human *ACTB* Gene Assay

## Abbreviations

CTCL	cutaneous T-cell lymphomas
AD	atopic dermatitis
CE	cornified envelope
mRNA	messenger ribonucleic acid
LELP-1	late cornified envelope-like proline-rich protein-1
SPRR1A	small proline-rich proteins 1A
SPRR1B	small proline-rich proteins 1B
SPRR3	small proline-rich proteins 3
RPTN	repetin
CRNN	cornulin
HRNR	hornerin
LOR	loricrin
FLG	filaggrin
FLG2	filaggrin 2
RT-PCR	real-time polymerase chain reaction
ELISA	enzyme-linked immunoabsorbent assay
PBS	phosphate buffered saline
